# Everybody's Page

**Published:** 1890-12-06

**Authors:** 


### EVERYBODY'S PAGE.

ERSKINE rather sacrificed his fellow-countrymen to his play upon words when he wrote :—

The French have taste in all they do,
Which we are quite without,
For nature, that to them gave *goût*,
To us gave only gout.

THE latest remedy suggested for the evils resulting from the sweating system is the establishment of a "Consumers' League," which, after due inquiry, will publish for the guidance of its members a list of "fair" employers in all branches of industry. It will be interesting to see what tradesmen who are not included in this list will have to say. Such a league may find itself in troubled waters.

THE favourite form of suicide in India is drowning, owing to the fact that this method of self-destruction does not involve personal mutilation. This form of suicide is probably resorted to sometimes not only in order to avoid mutilation, but with a view to propitiating the water-spirit by an act of self-sacrifice which has a fascination for some Eastern minds.

A REMARKABLE surgical operation is reported as having been made in the Charity Hospital, New York, where a portion of a living dog's foreleg has been grafted on to a boy's leg to take the place of a bone which is wanting. If the dog's limb unites with the boy's the flesh by which the animal is connected with the boy will then be cut. Psychologists are looking forward to the result with curiosity. Will animal propensities show undue prominence henceforward in the boy? The least the hospital can do for the dog is to present him with a wooden leg and a crutch.

THE chief clerk to one of the metropolitan police courts has recently pointed out how groundless are the charges of harsh sentences which are sometimes brought by persons who are not aware of the real facts of the case. A lad who was alleged to have been treated with monstrous harshness turns out to be a very old offender, and to have been convicted twice previously during this year. He confessed that he had work at £1 a-week, but he preferred not to work. If not a gentleman of culture, he is evidently one of varied tastes. His first theft this year was that of two iron harrows, his second was of boots, on the last occasion the "pains of hunger," which he might have satisfied by working at an honest livelihood, impelled him to take nine turnips from a market garden. As the magistrate's clerk wrote, "nine turnips were scarcely necessary for the alleviation of his hunger, unless he were looking for a particularly juicy one."

A. L. BICKNELL writes in *Murray's* an account of the condition of the working women in Paris, which seems to be as bad as that of the same class in London. A plain work needlewoman, if she works the entire day without interruption, may make the equivalent to an English shilling, but then during the *morte-saison*, which always lasts from two to three months, she can get no work at all. Sack-makers, bag-makers, and the lower grades of workers are paid still worse; by sixteen hours' continuous work it is possible to earn ninepence! The average rent paid for one room is 150 francs a-year, if a wretched, barely partitioned off closet is worthy of the name of room; and so the writer tells us those who have studied the question of female labour in Paris well and deeply are of opinion that those who must fight the problem of existence alone, and who are determined to keep "the white flower of a blameless life," must perish of utter starvation. "Cheapness" when investigated tells a pitiful tale.

THE report of the Chemist and the Engineer to the London County Council on their recent inspection of the Thames will come as a pleasant surprise to many Londoners. There appears to be a marked improvement by the outfall at Barking, which has at different times proved so obnoxious, and as soon as the Crossness works are completed a prospect is held out of further improvement in a state of affairs which has not been very desirable from a sanitary point of view.

A DISEASE, the nature of which is probably not as clearly understood in this country as in Germany, and which is commonly known to horse dealers by the unscientific name of Pink-eye, has been causing much trouble to the London Road Car Company. The disease first shows itself in the eye appearing very blood-shot; this symptom is followed by the appearance of large lumps all over the body which break out into open sores. It frequently proves fatal, in fact 17 horses died from it in the course of six days. It is supposed that a German horse is the cause of the outbreak.

THE Earl of Kilmorey evidently possesses a vein of humour. He congratulated the shareholders of the Automatic Photograph Company at the general meeting held last week, on the successful launching of the company, stating that the directors are highly pleased with the machines now being placed before the public, but that they had been horrified by the appearance of the first few pictures! He did not state the condition of mind of the people who were operated upon, possibly because the English language possesses no words capable of accomplishing this, or perhaps the directors themselves were the confiding victims.

THE evil influence on society in China of the modern Chinese novel is said to be exercising very deeply the minds of many Confucianists. The suggestions in this class of literature to young men to lead dissipated lives, and the representations that the act of killing a man is a noble one, produce disastrous results on public morality. This is but the revival of a similar evil which led to the introduction of very drastic remedies some thirty years ago. The pernicious influence of a certain class of novel is unfortunately known nearer home. Possibly Mr. Walter Besant's Academy of Letters, if it is ever established, will, like another academy, have a *hanging* committee.

